# Characteristics of patients returning to emergency departments in Naples, Italy

**DOI:** 10.1186/1472-6963-8-97

**Published:** 2008-05-02

**Authors:** Gabriella Di Giuseppe, Rossella Abbate, Luciana Albano, Paolo Marinelli, Italo F Angelillo, The Collaborative Research Group

**Affiliations:** 1 Department of Public, Clinical and Preventive Medicine, Second University of Naples, Naples, Italy

## Abstract

**Background:**

Crowding in hospital Emergency Departments (EDs) is a problem in several countries. We evaluated the number and characteristics of patients who make repeated visits to the EDs in Naples, Italy.

**Methods:**

All patients (≥ 16 years) who presented to the EDs of three randomly selected non-academic acute care public hospitals, within randomly selected week periods, were studied. The two outcomes of interest were the re-utilization, within 72 hours, of the ED and the number of visits in the previous year.

**Results:**

Of the 1430 sampled patients, 51.9% self-reported multiple visits in the previous year and 10.9% and 1.6% used the ED for 3 and ≥4 times, respectively. The number of visits in the previous year was significantly higher in those who live closer to hospital, with a more severe burden of overall comorbidity, and who were on pharmacological treatment. Overall, 72-hours return visits were found in 215 patients (15.8%). Patients were more likely to re-use within 72 hours the ED if younger, were not on pharmacological treatment, attended the ED more times in the previous year, were referred by a physician, arrived at the ED by car driven by other person, had problems of longer duration prior to arrival at the ED, had a surgical ED discharge diagnosis, and were admitted to the hospital.

**Conclusion:**

The data may assist policymakers in the development and implementation of protocols to track changes in the re-utilization of the ED for the high financial impact and for the benefit of the patients.

## Background

Crowding in hospital Emergency Departments (EDs) is a commonly observed problem in several industrialized countries [1-3]. Although the reasons and mechanisms are complex, the major factors are increasing volume of patients seeking medical care in ED services, lack of inpatient beds, and care for non-urgent conditions for patients who identify the ED as their usual site of care, notwithstanding the typical treatment of patients with serious illnesses or injuries. These patients typically do not receive any active ED intervention and could have managed adequately in other health care services, mainly the primary care facilities [4,5]. The decision to seek care through an ED is complex, and its explanation involves the consideration of many factors, such as patients' socio-demographic and economic characteristics, illness severity, and health service utilization behaviour.

The consequences of ED crowding include long waiting times which in turn causes delay in patient treatment and overwhelmed ED resources, and within hospital systems medical care provided by the ED represents a major component of the health care expenditures. Of particular interest is the group of patients who make many visits to ED within a brief period of time, since it is clear that frequent users consume a disproportionately large share of health care resources and hospital staff perceives them as burdensome to their workload.

Previous studies have examined the use of ED in Europe [6-8] and in the United States [9-12]. In Italy, there is a regionally-based National Health Service with an emergency medical system, providing services free of charge to all citizens, with two linked and interdependent components: pre-hospital care, provided in the community until the patient arrives at a facility capable of providing definitive care, and hospital care. No data are available in Italy and because is important to gather information on this topic from countries with this health care system, this present cross-sectional investigation sought to examine the number and characteristics of patients who make repeated visits to the EDs in Naples, Italy.

## Methods

Three EDs were randomly selected among the eight non-academic acute care public hospitals located in the geographic area of Naples (Italy). The selected hospitals have respectively 190 beds with 390,000 ED visits per year; 158 beds with 50,000 visits; and 150 beds with 74,000 visits. All patients aged 16 years or older who presented to the EDs, whether self or physician referred, from April 27 to December 17, 2006, within randomly assigned week periods were selected.

A medical interviewer, who had been previously trained and was not involved in health care, approached the patients who arrived at the ED for the current visit, indicated as index visit, after they had completed the ED registration process. After describing the purpose of the study, the interviewer collected for each patient who gave written informed consent to participate, with a brief questionnaire, the following information: socio-demographics characteristics (age, gender, marital status, educational level, living condition, employment status); distance the patient lived from the hospital; time, day of the week, and method of arrival; self-reported pre-admission performance-based measure of basic activities of daily living (BADL) using the Katz score [13]; comorbidity index of Charlson et al. [14]; pharmacological treatment(s); route of referral, symptom(s) from which the patient suffered before the presentation at the ED, duration of complaints prior to presentation; reasons for attending the ED; level of urgent care according to the Guidelines of the Canadian Emergency Department Triage and Acuity Scale [15]; and number of ED visits to acute care hospitals in the previous year. For each patient, the attending physician completed for the index visit a form about the consultation process (investigations, medical or surgical examinations, treatment received), ED discharge diagnosis, and final disposition. If a patient had already attended an ED in the previous year, the same information was obtained for every previous visit by personally asking the patient. When patient was unable to be interviewed because of his/her health status, the questionnaire was completed by relatives. A total of 27 patients were excluded because they were judged to have severe impairments, requiring immediate medical or surgical attention, that interfered with completion of the verbal assessment tools and no one else was available to provide the information.

The study was approved by the Ethics Committee of the Authors' Institution.

Non-traumatic injuries were classified anatomically (e.g., chest pain, abdominal pain) and by the most troubling symptom (e.g., shortness of breath, weakness, fever) whereas those traumatic were usually categorized by cause of injury (e.g., motor vehicle collision, fall, gunshot wound). The ED discharge diagnosis was classified according to the International Classification of Diseases (ICD)-10 and the patient was assigned to medical or surgical group on the basis of the advice of doing or not doing a surgical procedure.

The medical interviewers piloted the survey instrument with actual patients in the ED before the start of the study to evaluate validity, content, and clarity of each question and feedback was used to refine the survey instrument.

### Statistical analysis

A multivariate ordered logistic and logistic regression analyses were used to determine the independent association of the potential predictors' characteristics with the following outcomes of interest: profile of the patient who attend the ED according to the number of visits in the EDs in the previous year (Model 1) and index visit as a re-utilization of the ED within 72 hours from a previous ED visit in the same or other hospital (Model 2). Two procedures were performed for these analyses. First, statistical associations between various characteristics and the outcomes were assessed. Univariate analysis was conducted using appropriate test statistics and those characteristics associated with the outcome variables with a *p *value less or equal than 0.25 were included into the multivariate models. Second, multivariate models using backward elimination and stepwise selection of variables were developed and the significance level for variables entry into the final models was set at 0.2 and for removal at 0.4. The following explanatory variables were included in the models: age (continuous, in years), gender (male = 0, female = 1), marital status (single/separated/divorced/widowed = 0, married = 1), educational level (continuous, in years), number of other persons in the household (0 = 0, 1 = 1, 2 = 2, 3 = 3, ≥4 = 4), employment status (unemployed = 0, employed = 1), distance in kilometres between home and hospital (< 1 = 0, 1–10 = 1, > 10 = 2), total pre-admission performance-based measure of BADL (continuous), comorbidity index (continuous), and on pharmacological treatment (no = 0, yes = 1). The following variables, related to the index visit at the ED, were also included in Model 2: referral (self/relative = 0, physician = 1), mode of transportation (categorical, car driven by patient or on foot = 0, car driven by other person = 1, ambulance = 2), arrival time (2.00 PM-7.59 AM = 0, 8.00 AM-1.59 PM = 1), day of the week of attending (monday-friday = 0, saturday-sunday = 1), duration of presenting problem prior to arrival, in hours (< 1 = 0, 1–24 = 1, > 24 = 2), principal reason for attending (non-traumatic injury = 0, traumatic injury = 1), therapeutic procedure(s) received (no = 0, yes = 1), diagnostic investigation(s) received (no = 0, yes = 1), physician(s) consultation(s) received (no = 0, yes = 1), level of urgent care (categorical, non urgent = 0, urgent = 1, emergency = 2), ED discharge diagnosis (medical = 0, surgical = 1), hospital admission after the visit (no = 0, yes = 1), therapy prescription (no = 0, yes = 1), physician examination(s) prescription (no = 0, yes = 1), and number of ED visits in the previous year (1 = 1, 2 = 2, 3 = 3, ≥4 = 4). Logistic regression analysis was conducted to calculate the odds ratios (ORs) and their corresponding 95% confidence intervals (CIs) for the association between independent variables and outcome. A two-tailed *p*-value of less or equal than 0.05 was the criterion for statistical significance. All analyses were conducted using the Stata version 8.1 software program [16].

## Results

Of the 1430 patients selected, a total of 1360 agreed to participate for a response rate of 96.9% and for 14.2% of them the data was collected by proxy. Table 1 shows the characteristics of patients attending the ED according to visit frequency. More than half were male, the mean age was 46.7 years, the vast majority attended the ED during weekdays, the time of arrival was between 8 AM and 2 PM, three-quarters of visits were for non-traumatic reasons although the rate of transports for car driven by other person was the highest, only 8.1% were referred by physicians, and almost eight of ten cases were triaged as emergent or urgent.

**Table 1:** Characteristics of patients attending the Emergency Department (ED) by visit frequency

	All patients (n = 1360)		Patients with more than one visit in the previous year				Patients with the index visit within 72 hours after a previous one			
				
			No (n = 654)		Yes (n = 706)		No (n = 1145)		Yes (n = 215)	
	n	%	n	%	n	%	n	%	n	%

*Gender*										
Male	771	56.7	377	57.6	394	55.8	645	56.3	126	58.6
Female	589	43.3	277	42.4	312	44.2	500	43.7	89	41.4
							χ^2 ^= 0.38, 1 df, *p *= 0.54			

*Age (years)*	46.7 ± 18.8 (16–97)*		43.8 ± 17.9 (16–95)*		49.3 ± 19.2 (16–97)*		47.2 ± 18.8 (16–97)*		44.2 ± 18.5 (16–88)*	
							*t *test = 2.14, df = 1358, *p *= 0.033			

*Marital status*										
Married	865	63.6	413	63.1	452	64	738	64.4	127	59.1
Other	495	36.4	241	36.9	254	36	407	35.6	88	40.9
							χ^2 ^= 2.27, 1 df, *p *= 0.13			

*Educational level (years of schooling)*	8.1 ± 4 (0–23)*		8.7 ± 4.2 (0–23)*		7.6 ± 3.7 (0–20)*		8.1 ± 4 (0–23)*		8.1 ± 3.8 (0–18)*	
							*t *test = 0.18, df = 1358, *p *= 0.86			

*Number of other persons in the household*										
0	115	8.5	45	6.9	70	9.9	99	8.6	16	7.4
1	221	16.2	88	13.5	133	18.8	184	16.1	37	17.2
2	298	21.9	165	25.2	133	18.8	254	22.2	44	20.5
3	359	26.4	182	27.8	177	25.1	292	25.5	67	31.2
4	367	27	174	26.6	193	27.4	316	27.6	51	23.7
							χ^2 ^= 3.9, 4 df, *p *= 0.42			

*Employment status*										
Unemployed	811	59.6	355	54.3	456	64.6	678	59.2	133	61.9
Employed	549	40.4	299	45.7	250	35.4	467	40.8	82	38.1
							χ^2 ^= 0.53, 1 df, *p *= 0.47			

*Distance from patients' home to hospital (Km)*										
< 1	819	60.2	372	56.9	447	63.3	686	59.9	133	61.9
1–10	332	24.4	169	25.8	163	23.1	278	24.3	54	25.1
> 10	209	15.4	113	17.3	96	13.6	181	15.8	28	13
							χ^2 ^= 1.08, 2 df, *p *= 0.58			

*Day of the week of attending the ED*										
Monday-Friday	1193	87.7	598	91.4	595	84.3	1015	88.6	178	82.8
Saturday-Sunday	167	12.3	56	8.6	111	15.7	130	11.4	37	17.2
							χ^2 ^= 5.76, 1 df, *p *= 0.016			

*Arrival time at the ED*										
8.00 AM-1.59 PM	796	58.5	389	59.5	407	57.6	668	58.3	128	59.5
2.00 PM-7.59 AM	564	41.5	265	40.5	299	42.4	477	41.7	87	40.5
							χ^2 ^= 0.11, 1 df, *p *= 0.74			

*Mode of transportation to the ED*										
Ambulance	82	6	43	6.6	39	5.5	76	6.6	6	2.8
Car driven by other person	1011	74.4	479	73.2	532	75.4	844	73.7	167	77.7
Car driven by patient/On foot	267	19.6	132	20.2	135	19.1	225	19.7	42	19.5
							χ^2 ^= 4.83, 2 df, *p *= 0.09			

*On pharmacological treatment*										
Yes	513	37.7	186	28.4	327	46.3	444	38.8	69	32.1
No	847	62.3	468	71.6	379	53.7	701	61.2	146	67.9
							χ^2 ^= 3.44, 1 df, *p *= 0.06			

*Charlson et al. comorbidity index*	0.5 ± 1 (0–8)*		0.3 ± 0.8 (0–6)*		0.6 ± 1.1 (0–8)*		0.5 ± 1 (0–8)*		0.4 ± 1 (0–6)*	
							*t *test = 1.05, df = 1358, *p *= 0.29			

*Basic Activities of Daily Living score*	5.7 ± 1.1 (0–6)*		5.9 ± 0.8 (0–6)*		5.6 ± 1.3 (0–6)*		5.7 ± 1.1 (0–6)*		5.7 ± 1.1 (0–6)*	
							*t *test = -0.28, df = 1358, *p *= 0.78			

*Referral to the ED*										
Physician	110	8.1	45	6.9	65	9.2	75	6.6	35	16.3
Self/relatives	1250	91.9	609	93.1	641	90.8	1070	93.4	180	83.7
							χ^2 ^= 23.05, 1 df, *p *< 0.001			

*Principal reason for attending the ED*										
Non-traumatic injuries	998	73.4	418	63.9	580	82.1	835	72.9	163	75.8
Traumatic injuries	362	26.6	236	36.1	126	17.9	310	27.1	52	24.2
							χ^2 ^= 0.77, 1 df, *p *= 0.38			

*Duration of presenting problem prior to arrive at the ED (hours)*										
< 1	474	34.9	272	41.6	202	28.6	431	37.6	43	20
1–24	610	44.8	288	44	322	45.6	499	43.6	111	51.6
> 24	276	20.3	94	14.4	182	25.8	215	18.8	61	28.4
							χ^2 ^= 26.99, 2 df, *p *< 0.001			

*Diagnostic investigation(s) received at the ED*										
Yes	758	55.7	376	57.5	382	54.1	656	57.3	102	47.4
No	602	44.3	278	42.5	324	45.9	489	42.7	113	52.6
							χ^2 ^= 7.01, 1 df, *p *= 0.008			

*Therapeutic procedure(s) received at the ED*										
Yes	711	52.3	327	50	384	54.4	615	53.7	96	44.7
No	649	47.7	327	50	322	45.6	530	46.3	119	55.3
							χ^2 ^= 5.96, 1 df, *p *= 0.015			

*Physician consultation(s) received at the ED*										
Yes	701	51.5	302	46.2	399	56.5	574	50.1	127	59.1
No	659	48.5	352	53.8	307	43.5	571	49.9	88	40.9
							χ^2 ^= 5.79, 1 df, *p *= 0.016			

*Level of urgent care*										
Emergency	428	31.5	219	33.5	209	29.6	363	31.7	65	30.2
Urgent	654	48.1	308	47.1	346	49	554	48.4	100	46.5
Non-urgent	278	20.4	127	19.4	151	21.4	228	19.9	50	23.3
							χ^2 ^= 1.25, 2 df, *p *= 0.54			

*ED discharge diagnosis*										
Medical	691	50.8	284	43.4	407	57.6	595	52	96	44.7
Surgical	669	49.2	370	56.6	299	42.4	550	48	119	55.3
							χ^2 ^= 3.87, 1 df, *p *= 0.049			

*Hospital admission after ED visit*										
Yes	203	14.9	80	12.2	123	17.4	162	14.2	41	19.1
No	1157	85.1	574	87.8	583	82.6	983	85.8	174	80.9
							χ^2 ^= 3.45, 1 df, *p *= 0.06			

*Prescription of therapy*										
Yes	772	56.8	407	62.2	365	51.7	672	58.7	100	46.5
No	588	43.2	247	37.8	341	48.3	473	41.3	115	53.5
							χ^2 ^= 10.94, 1 df, *p *= 0.001			

*Prescription of physician examination(s)*										
Yes	511	37.6	241	36.9	270	38.2	428	37.4	83	38.6
No	849	62.4	413	63.1	436	61.8	717	62.6	132	61.4
							χ^2 ^= 0.12, 1 df, *p *= 0.73			

*Number of ED visits in the previous year*										
1	654	48.1	-	-	-	-	654	57.1	-	-
2	536	39.4	-	-	536	75.9	376	32.8	160	74.4
3	148	10.9	-	-	148	21	106	9.3	42	19.5
≥4	22	1.6	-	-	22	3.1	9	0.8	13	6.1
							χ^2 ^= 250.74, 3 df, *p *< 0.001			

*Mean ± Standard deviation (Range)

A total of 706 patients (51.9%) self-reported multiple visits to the ED in the previous year and 10.9% and 1.6% used the ED three and four or more times, respectively. Overall, 72-hours return visits were found in 215 patients (15.8%) and respectively 11 (5.1%) and 7 (3.3%) of them returned three and four times in the 72 hours. In the first model the dependent variable was the number of ED visits in the previous year. Results of the stepwise multiple ordered logistic regression analysis showed that the number of visits was significantly higher in patients with shorter distance from home to hospital, in those with a more severe burden of overall comorbidity, and in those on pharmacological treatment (Model 1 in Table 2).

**Table 2:** Results of the multivariate ordered logistic (1) and logistic (2) regression models

Variable	Coeff.	SE	z	*p*
**Model 1. **Number of Emergency Department visits in the previous year				
Log likelihood = -1347.38, χ^2 ^= 99.01 (6 df), *p *< 0.0001				
On pharmacological treatment	0.51	0.12	4.13	< 0.001
Comorbidity index	0.17	0.07	2.48	0.013
Distance from patients' home to hospital:				
> 10 Km*	-	-	-	-
< 1 Km	0.27	0.11	2.49	0.013
Educational level	-0.03	0.15	-1.82	0.069
Employment status	-0.2	0.11	-1.76	0.079
Basic Activities of Daily Living score	-0.09	0.05	-1.71	0.088

Variable	OR	SE	95% CI	*p*

**Model 2. **Index visit as a re-utilization of the Emergency Department after 72 hours from a previous Emergency Department visit				
Log likelihood = -451.26, χ^2 ^= 284.73 (15 df), *p *< 0.0001				
Number of Emergency Department visits in the previous year	5.0	0.65	3.89–6.45	< 0.001
Referral	3.12	0.86	1.81–5.35	< 0.001
Duration of presenting problem prior to arrive at the Emergency Department:				
< 1 hour*	1.0			
1–24 hours	1.91	0.42	1.25–2.94	0.003
> 24 hours	2.06	0.51	1.27–3.37	0.004
On pharmacological treatment	0.56	0.13	0.36–0.86	0.009
Mode of transportation to the Emergency Department:				
Ambulance*	1.0			
Car driven by other person	3.22	1.61	1.21–8.58	0.019
Car driven by patient/On foot	2.54	1.36	0.89–7.24	0.08
Emergency Department discharge diagnosis	1.56	0.3	1.07–2.28	0.022
Hospital admission after Emergency Department visit	1.82	0.51	1.06–3.14	0.031
Age	0.99	0.01	0.98–0.99	0.039
Therapeutic procedure(s) received at the Emergency Department	0.69	0.14	0.47–1.01	0.057
Physician consultation(s) received at the Emergency Department	0.72	0.14	0.49–1.07	0.1
Day of the week of attending the Emergency Department	1.42	0.34	0.89–2.26	0.14
Prescription of therapy	0.8	0.15	0.55–1.16	0.24
Investigation(s) received at the Emergency Department	0.82	0.16	0.57–1.19	0.31

*Reference category

After multivariate logistic adjustments, the results regarding the re-utilization of the ED within 72 hours from a previous ED visit were partially in agreement with those from the unadjusted associations. Of the personal patient characteristics, only age and current pharmacological treatment were significantly associated with the outcome of interest because those younger (OR = 0.99; 95% CI 0.98–0.99) and those not on treatment (OR = 0.56; 95% CI 0.36–0.86) were more likely to attend the ED more than once in 72 hours. The odds of more than one visit in 72 hours increased by 5 times for patients who have attended more times the ED in the previous year (95% CI 3.89–6.45). How the patient arrived at the ED was also a significant predictor, since, when transportation by ambulance was chosen as reference category, the odds of more than one visit increased significantly by about 3 times for those who arrived by car driven by another person (95% CI 1.21–8.58) as compared with patients who were transported by ambulance. The clinical characteristics of the patients who attended more than once in 72 hours differed from those who attend only one time. Indeed, those with problems of longer duration prior to arrival at the ED (OR = 1.91; 95% CI 1.25–2.94; OR = 2.06; 95% CI 1.27–3.37) and with a surgical ED discharge diagnosis (OR = 1.56; 95% CI 1.07–2.28) attended more than once. Finally, referral and final disposition were also strong predictors of more than one ED visit in 72 hours. Indeed, patient referred by a physician was 3.12 times more likely to return compared to self or relatives referred (95% CI 1.81–5.35) and those who return were almost twice as likely to be hospital admitted, including critical care units (95% CI 1.06–3.14) (Model 2 in Table 2).

## Discussion

This report describes the magnitude and frequency of adult patients who make repeated visits to the EDs as well the predictors of such use in an area of Italy.

Comparison with other studies in the literature on re-utilization of the ED by adult patients is difficult, because there are wide differences in health care delivery system of the countries and in study design such as, for example, format of the source of data collection, time periods, and age groupings of the study population. In this study, 51.9% of patients attended an ED for more than one visit in a year and 10.9% and 1.6% attended 3 and ≥4 times. Studies performed in countries with a health care system with universal access showed that in a University public Hospital in Sweden 26.9% of patients aged 15 and above sought care more than once in the ED in a year and 4% made ≥4 visits [16]; in an urban UK city over one year 3.5% and 2.2% patients made 3 and ≥4 attendances, respectively [8]. Our value was considerably higher than the 31.4%, collected from a statewide dataset in the United States during one fiscal year in patients of all age groups [12]. Our results were considerably lower than those observed in previously published reports. Indeed, in a National Survey of America's Families, 3% of individuals made ≥3 visits in a year [9], in a US national population-based data source, 8% of patients aged 18 and older made ≥4 visits in a year [2], 4.5% in a statewide database [20], and 6% in a large rural academic medical center [21]. Our value that nearly one fifth (15.8%) of the total attendances re-utilized an ED more than once in 72-hours was generally higher than those observed in the United States within a calendar year with values of 7.1% in an already mentioned study [21], of 3.1% to 3.7% in a nationally representative sample [12], and of 0.27% to 0.93%, in two years, in a University Medical Center [10].

The investigation of the relationship between several characteristics and outcomes of interest contribute to identifying those patients who frequently re-use ED services in Italy and yielded several interesting findings. Some of the findings are remarkable in that they have important implications for health care workers in ED and for administrators who design systems to improve efficiency and quality of emergency care. The information of this study enables clinicians to identify patients at high risk of ED re-utilization and some of these findings confirmed those of other studies, particularly that patients in fair/poor health were more likely to be frequent users [1,2,9,22,23]. Of note, we further found that more than one ED re-utilization within 72 hours was associated with the mode of transportation since it was significantly higher for patients arriving by independent means. A previous study has suggested that patients with potentially more serious illness or injury were more likely to be transported by ambulance [24]. Moreover, a significantly higher proportion of patients referred by physicians, usually in primary care, re-used the ED within 72 hours compared with those who were self or relatives referred, suggesting a degree of clinical selection prior to attendance. This is also confirmed by the fact that no significant association has been observed between re-utilization of the ED within 72 hours and the level of urgent care. Additionally, this result is in accordance with a study conducted by some of us [4]. This inference is not surprising at all and may also be attributed to our health care system. Finally, our results also showed that those who use the ED more frequently are more likely to live closer to the hospital. Such association may suggest that at least some patients may be using the ED services for the convenience of having the hospital near home and for routine care, a finding consistent with a previous study [19]. This study suggests the need to improve communication and coordination between EDs and primary care physicians and to provide adequate patient and family education about the health care delivery system.

In interpreting the findings, potential methodological limitations should be considered. The study was conducted during the period April-December and a potential bias for seasonal variation in utilization may be present. However, in studies, conducted in a one year period in a similar geographic area, no differences have been observed according to seasons [17-19] and since differences in this one were not aspect, we are confident that there is not such potential bias. Data regarding previous ED visits were directly collected by interviewing the patient, and we cannot be absolutely certain about validity of the responses. Therefore, the results might be influenced by a recollection bias, because patients were asked about other ED visits that may have occurred up to one year before and they may have been less likely to remember with an underestimation of the true proportion of re-visits. We were willing to accept this limitation, mainly because we believe that survey respondents correctly recall this information and because the principal focus was to assess the extent of patients who were making repeated visits to the ED within 72 hours from the index visit and the predictors of this re-utilization. Despite these limitations, there are several important strengths of the study. First, this is one of the few studies that collected data by interviewing patients at the ED and not by retrospective medical record reviews, with the possibility of gathering more detailed information. Second, including data from various institutions, potential biases inherent in the study of patients from a single institution have been avoided, and these data may be generalisable to all EDs. Third, data were collected over an adequate period of time. Fourth, a large number of patients agreed to participate and a such high response rate is not uncommon in cross-sectional surveys on patients attending emergency health services [7,25-28]. Fifth, multivariable analyses allowed for adjustment of several covariates.

## Conclusion

In summary, the policy and management implications of these findings into the complex debate of crowding in hospital EDs are clear. The challenge for the future is that policy makers in hospital and in public health arenas should develop and implement protocols in order to track changes, not just in view of the high financial impact, but also for the benefit of the population.

## Competing interests

The authors declare that they have no competing interests.

## Authors' contributions

GDG and RA participated in the design of the study, collected the data, and contributed to the data analysis and interpretation; LA collected the data and contributed to the data analysis; PM participated in the design of the study and contributed to the interpretation of the data; IFA, the principal investigator, designed the study, was responsible for the data collection, statistical analysis and interpretation, and wrote the article. All authors have read and approved the final version of the manuscript.

## Pre-publication history

The pre-publication history for this paper can be accessed here:


